# Reduced gastric acidity, proton pump inhibitors and increased severity of COVID-19 infections

**DOI:** 10.1186/s13054-021-03497-6

**Published:** 2021-02-18

**Authors:** Elizabeth Price, David F. Treacher

**Affiliations:** 1 London, UK; 2 Consultant in Intensive Care and Critical Care Medicine, Guy’s and St Thomas’ NHS Foundation Trust, London, UK

Dear Editor

There are now articles linking the use of proton pump inhibitors (PPIs) to an increased risk of more severe COVID-19 infections [1, 2]. As discussed in our research letter [3], this is not surprising because these viruses can be swallowed and may spread via the gastrointestinal tract as well as the respiratory tract. Gastric acid is a partial barrier which restricts the entry of SARS-CoV-2 viruses into the rest of the gastrointestinal tract. This acid barrier is removed by a single dose of a PPI, as it raises the gastric pH from its usual level of 1.5 -3.5 to over 6.0. At this less acid pH, these viruses are not inactivated [3, 4]. This may be part of the reason young people and children rarely get a severe COVID-19 illness, as this age group usually has good gastric acidity.

For ventilated patients, as well as the PPI risk, continuous nasogastric feeding regimes may present a risk of a more severe COVID-19 illness. For patients on these continuous regimes, the gastric pH will be around 4.0 or 5.0. At this pH, SARS-CoV-2 viruses will not be inactivated [3, 4]. In contrast, intermittent nasogastric feeding will allow the gastric pH to fall to < 2.0 between periods of feeding. At this more acid pH level, the viruses could be inactivated.

During the present pandemic, it would be useful if a risk–benefit assessment could be carried out for patients on PPIs. A histamine-2 receptor antagonist (sometimes specified as famotidine) has been mentioned as a possible alternative but further review is needed [2, 5]. In addition, maintaining gastric acidity in ventilated COVID-19 patients by intermittent nasogastric feeding, could be considered where possible.

## Data Availability

Not applicable.
